# XAFS measurement system using undulator synchronized on-the-fly monochromator scan at SPring-8 BL36XU

**DOI:** 10.1107/S160057752600768X

**Published:** 2026-08-26

**Authors:** Nozomu Ishiguro, Tomoya Uruga, Takuma Kaneko, Takashi Tanaka, Yasumasa Joti, Koji Motomura, Hiroshi Yamazaki, Yasunori Senba, Haruhiko Ohashi, Makina Yabashi, Yukio Takahashi

**Affiliations:** a International Center for Synchrotron Radiation Innovation Smart (SRIS) Tohoku University Katahira 2-1-1 Aoba-ku Sendai980-8577 Japan; b RIKEN SPring-8 Center, 1-1-1 Koto, Sayo, Hyogo679-5148, Japan; c Japan Synchrotron Radiation Institute (JASRI) SPring-8 1-1-1 Koto Sayo Hyogo679-5198 Japan; d Graduate School of Human and Environmental Studies Kyoto University Yoshida Nihonmatsu-cho Sakyo-ku Kyoto606-8501 Japan; e Institute of Multidisciplinary Research for Advanced Materials (IMRAM) Tohoku University Katahira 2-1-1 Aoba-ku Sendai980-8577 Japan; f Institute for Materials Research (IMR) Tohoku University Katahira 2-1-1 Aoba-ku Sendai980-8577 Japan; ESRF – The European Synchrotron, France

**Keywords:** quick XAFS, undulator–monochromator synchronization, on-the-fly scans, EXAFS

## Abstract

In X-ray absorption fine structure (XAFS) measurements at undulator beamlines, it has been difficult to simultaneously achieve both spectral time resolution and high X-ray flux that fully utilizes the optical performance of the beamline. To overcome this challenge, we developed a synchronization architecture for undulator beamlines, in which the undulator gap serves as the master axis while a compact channel-cut monochromator follows a pre-calculated nonlinear trajectory. In contrast to conventional feedback approaches, in which the monochromator serves as the master, the undulator gap is used as the master axis, enabling fast and flexible nonlinear synchronization with the compact channel-cut monochromator. Transmission and fluorescence quick-XAFS (QXAFS) measurements were successfully performed at the Ni *K* edge in the extended X-ray absorption fine structure (EXAFS) region within 6.4 s. The incident photon flux was six times higher than that of conventional QXAFS using a tapered and fixed undulator gap at the same acquisition time, demonstrating that the proposed synchronization architecture enables high-flux EXAFS measurements that were previously difficult to achieve with undulator-based QXAFS.

## Abstract 2

Nonlinear scanning of a compact channel-cut monochromator synchronized with an undulator-gap movement system enables fast high-flux X-ray absorption fine structure measurements.

## 1. Introduction

X-ray absorption fine structure (XAFS) is a highly powerful technique capable of performing element-selective electronic state/local structure analysis on various sample states, such as crystalline/amorphous, trace/dilute-concentration samples, solid/liquid/gas and *in situ/operando*. The XAFS spectrum includes two sections: X-ray absorption near-edge structure (XANES) and extended X-ray absorption fine structure (EXAFS). The XANES region is located around 50 eV from the absorption edge and reflects the local electron structure around the absorbed atom (*e.g.* valence or electron orbital symmetry). The EXAFS region, located at a higher-energy region, exhibits oscillation structures resulting from the scattering of photoelectrons with surrounding atoms from the absorption center. In general, a higher X-ray flux is required to analyze EXAFS oscillations and the local coordination structures of the sample material because their signals become exponentially weaker within the *k* (photoelectron momentum) range. XAFS has also evolved beyond simple spectroscopic measurements toward multidimensional and multifaceted approaches. For example, high-energy-resolution fluorescence detected X-ray absorption near-edge structure (HERFD-XANES) and resonant inelastic X-ray scattering (RIXS) measurements, detecting the emitted X-rays with high energy resolution using a spectroscopic crystal, reduce the influence of lifetime broadening, yielding sharper XANES spectra, enabling the detection of low-energy excitations within materials, such as crystal-field and inter-orbital excitations (*e.g.d*–*d*, *f*–*f*), charge-transfer excitations, magnetic excitations (magnons), and phonon excitations. X-ray spectro-microscopy combined with imaging techniques [*e.g.* transmission X-ray microscopy (TXM), scanning transmission X-ray microscopy (STXM), ptychography, *etc*.] provides nonuniform micro/nano-structures of target samples with their local electronic and coordination structures. Time-resolved XAFS with single measurements is essential for tracking sample changes during transient phenomena such as catalytic reactions or battery charging. If such advanced measurements can be performed at higher flux levels, it will be possible to extract meaningful information from the analysis of finer structures revealed by XAFS spectra — particularly the local coordination structures obtained via EXAFS — and this is likely to provide important insights into structural changes during charge–discharge reactions in materials such as those used in amorphous batteries.

Among time-resolved measurement techniques, the quick-XAFS (QXAFS) method enables fast XAFS measurements in ∼1 min or less by performing on-the-fly monochromator scans (Nachtegaal *et al.*, 2016 ▸). Moreover, the technique has been incorporated in several beamlines. Specifically, time-resolved super QXAFS measurements down to 20 ms have been achieved by rapidly scanning a channel-cut monochromator (CCM) with a simplified single-drive axis and are being used for *operando* time-resolved studies (Ishiguro *et al.*, 2014 ▸, 2016 ▸; Ozawa *et al.*, 2018 ▸). Similar optics are also being utilized at the Swiss Light Source (SLS), Super-XAS (Müller *et al.*, 2016 ▸) and PETRA-III (Caliebe *et al.*, 2019 ▸; Bornmann *et al.*, 2019 ▸). Furthermore, it has been demonstrated that performing QXAFS measurements at each computed tomography (CT) measurement angle yields higher-quality chemical-state imaging in 3D XAFS-CT measurements than acquiring CT data at the X-ray energy of each XAFS measurement (Matsui *et al.*, 2022 ▸). This is because the latter method extracts XAFS data for each pixel from CT data at each energy, making it susceptible to effects such as positional drift during sample rotation. In contrast, the former method minimizes these effects while ensuring high reproducibility of the incident X-ray energy by correcting the monochromator angle using encoder values.

Although the QXAFS method has advantages, care must be taken with the incident X-ray bandwidth when using it on an undulator beamline. The energy bandwidth of an undulator beamline is ∼0.1–1%; when the undulator gap is stationary, it is manageable in the XANES region (with a measurement range of ∼50 eV). However, sufficient intensity cannot be obtained when extended to the EXAFS region (with a measurement range of 1 keV or more). In contrast, beamlines capable of high-speed super QXAFS measurements using an undulator light source, including reports by Ishiguro *et al.* (2014 ▸, 2016 ▸), are equipped with a gap taper function that allows the energy bandwidth to exceed 1 keV, enabling time-resolved QXAFS measurements that include the EXAFS region (Caliebe *et al.*, 2019 ▸; Bornmann *et al.*, 2019 ▸). However, the photon flux drops from 1/5 to 1/10 of the peak in the energy band without tapering.

Hence, synchronization between the monochromator and undulator-gap scans is required to achieve both high speed and high flux in XAFS measurements. The relationship between the undulator gap (Δ*g*_u_), peak energy (*E*_peak_) and monochromator angle (θ_B_) is given by the following equations,
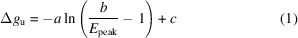
and
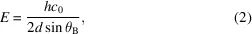
where *a*, *b* and *c* are constant values, *h* denotes Planck’s constant, *c*_0_ denotes the vacuum light speed, and *d* is the monochromator spacing distance [see Appendix *A*[App appa] for a detailed derivation of equation (1)]. Thus, the synchronized scanning of the undulator gap and the monochromator is nonlinear, requiring both high speed and high precision.

The development of undulator–monochromator synchronized scanning has been reported at several facilities. At the SLS X11MA SIM beamline, synchronization was achieved by updating and tracking the undulator-gap setting every 0.5 s based on the planar grating monochromator (PGM) readback during constant-speed scanning. Using this method, they maintained the PGM and undulator-gap readback values within ∼1.2 ± 0.3 eV at all times. In addition, they successfully measured the Co *L*_III_ edge (760–820 eV) range in 120 s (Krempaský *et al.*, 2010 ▸). At the Soleil TEMPO beamline, the SUMS system converts the multi-axis control of the undulator gap and monochromator into physical units in real time. It links them, thereby achieving undulator–monochromator synchronized on-the-fly scanning (Izquierdo *et al.*, 2012 ▸). A similar mechanism is used for undulator–monochromator synchronized scanning in the hard X-ray region. For example, at the PETRA-III P06 beamline, synchronized control with double-crystal monochromator (DCM) control as the master and undulator-gap scanning as the slave keeps the difference in undulator-gap peak energy consistently within 2 eV, enabling measurements of a 1 keV range in 30–120 s (Chernikov *et al.*, 2016 ▸). At the NSLS-II 5-ID SRX beamline, linear encoders and rotary encoders are installed on the control axes of the in-vacuum undulator (IVU) and horizontal DCM, respectively. These are connected via optical fiber to enable communication between them, and by updating the control of each servo motor every 65 kHz, advanced synchronization control is achieved. This mechanism has enabled the successful measurement of EXAFS spectra with a resolution of 10 eV s^−1^, or 1–120 s (Hidas *et al.*, 2022 ▸). A common feature of the examples already reported is the concept of using feedback to track the undulator gap while the monochromator performs constant-speed scanning. PGMs and DCMs require nonlinear simultaneous multi-axis control to vary the X-ray photon energy, and it has been difficult to achieve further speed increases under current conditions. Consequently, the achievable spectral time resolution is limited to tens of seconds to sub-minutes.

In this study, we investigated feedforward synchronized scanning in which the undulator gap serves as the master and the monochromator angle serves as the slave. The proposed architecture differs fundamentally from previously reported feedback-based synchronization systems in that the undulator, rather than the monochromator, defines the energy trajectory. This is because (1) increasing the complexity of the undulator-gap scanning mechanism affects the correction of fluctuations in the electron-beam trajectory within the storage ring (steering coil correction), which could lead to instability in the operation of the entire facility; therefore, it should be kept within the scope of standard drive methods; and (2) the compact CCM (c-CCM) at BL36XU is lightweight and capable of free-curve control in a single-axis operation, enabling it to track even complex behavior. This new measurement mechanism enabled spectral acquisition times that were several times faster than those reported previously.

## 2. Experimental section

### 2.1. Beamline optics and synchronization control of undulator and monochromator

**Figure 1:**
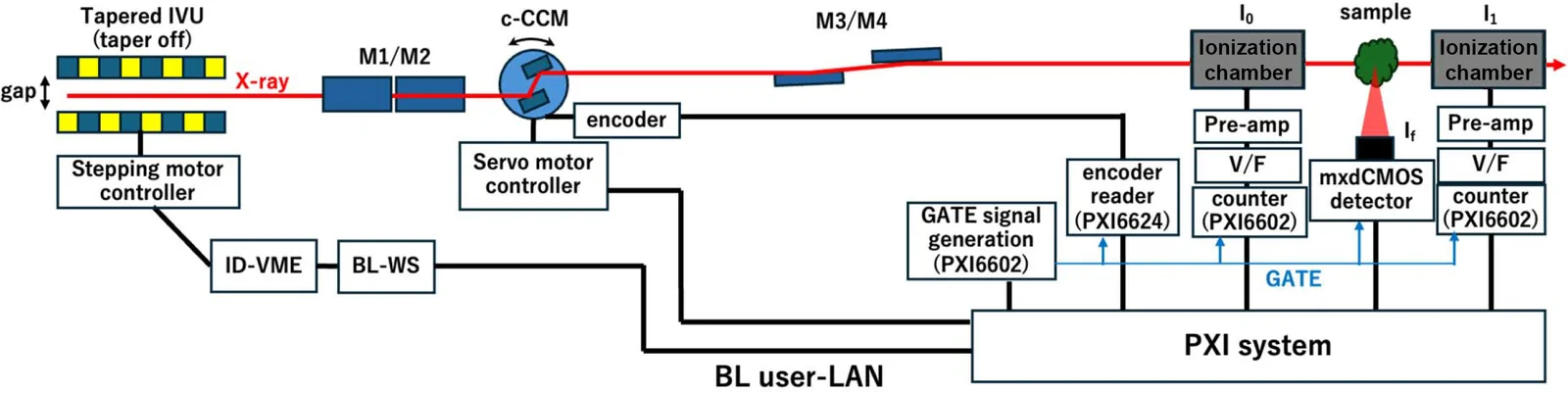
Schematic diagram of the undulator-gap synchronized monochromator on-the-fly scanning system at SPring-8 BL36XU.

Fig. 1 ▸ shows a schematic diagram of the optical system and the measurement and control system for on-the-fly scanning QXAFS with undulator-gap synchronization at the SPring-8 BL36XU beamline. The BL36XU optical system consists of a tapered undulator (ID), horizontal-deflection mirrors (M1/M2), two c-CCMs [Si(111)/Si(220)], vertical-deflection mirrors (M3/M4) and four transport-channel slits (TC slits 1–4) (Uruga *et al.*, 2019 ▸). These transfer-channel optical elements, excluding the c-CCMs, are controlled via the BL-LAN network through the beamline workstation (BL-WS) and BL-VME/ID-VME servers. The c-CCMs are connected to a direct-drive servo motor and are controlled by a dedicated controller via the BL-LAN. The angles of the c-CCMs can be read in real time via encoders. Measurement control at BL36XU is performed using a PXI system [PXIe-8881, National Instruments (NI)]. Signal detection was performed synchronously using a pulse signal emitted from a counter/timer board (PXI-6602, NI) as a common GATE signal: the counter board (PXI-6624, NI) for reading encoder values (monochromator angle) and the PXI-6602 for the *I*_0_/*I*_1_ ion chamber, as well as the mxdCMOS fluorescent detector (Kudo *et al.*, 2026 ▸), were measured synchronously. For partial fluorescence yield (PFY) mode measurement, elastic scattering X-rays were filtered by *Z* − 1 (Co) filter and Soller slits, and an energy range of 7.0–8.0 keV (Ni *K*α) was extracted as fluorescent signals (*I*_f_) by single-channel analyzer.

### 2.2. Undulator-gap synchronized QXAFS measurements

**Figure 2:**
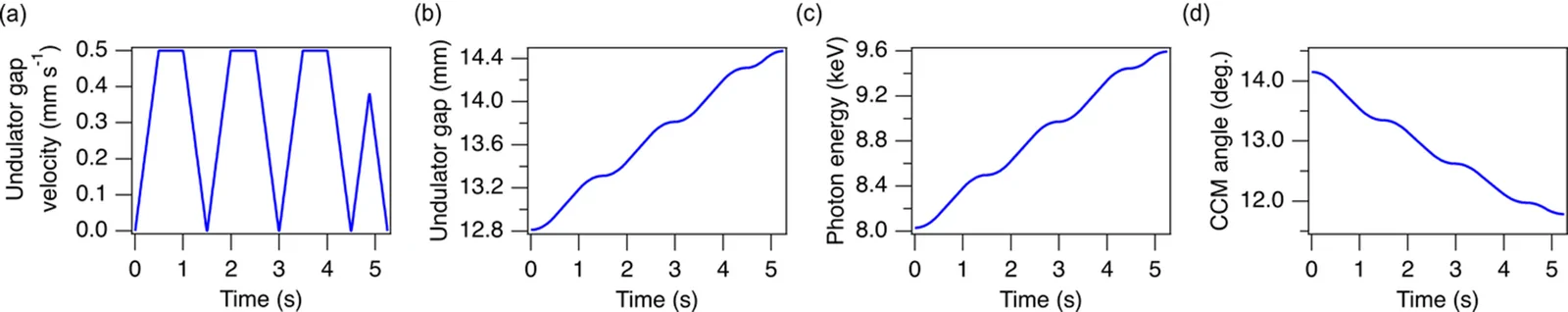
Calculated time profiles of (*a*) undulator-gap scan rate, (*b*) undulator gap, (*c*) photon energy of first-order X-ray emitted from the undulator and (*d*) the Si(111) c-CCM angle, at an undulator-gap synchronized monochromator on-the-fly scanning system.

The EXAFS region of the Ni *K* edge (8.03–9.57 keV) was selected as the measurement range, and Ni foil and Pt_3_Ni/C electrocatalysts in the membrane electrode assembly (MEA) of the polymer electrolyte fuel cell (PEFC) were chosen as the samples for transmission and PFY modes, respectively. For the common GATE signal, a pulse signal with a 99.9% duty cycle and frequencies of 1 kHz (transmission mode) or 700 Hz (PFY mode) was used to acquire detector and monochromator angle encoder values. Currently, the SPring-8 undulator gap is moved using a trapezoidal drive consisting of acceleration, constant speed and deceleration in increments of 0.5 mm [Fig. 2 ▸(*a*)]. Therefore, during the on-the-fly measurement from 8.03 to 9.57 keV, the undulator gap changes in a step-like manner over 5.4 s, as shown in Fig. 2 ▸(*b*). From the undulator-gap curve shown in Fig. 2 ▸(*b*), the peak energy of the primary undulator beam [Fig. 2 ▸(*c*)] can be obtained using equation (1). In contrast, the Si(111) monochromator crystal angle [Fig. 2 ▸(*d*)] can be obtained using equation (2). By registering the curve shown in Fig. 2 ▸(*d*) in the c-CCM servo motor controller, we achieved feedforward on-the-fly synchronized scanning of the undulator gap and monochromator. However, in actual measurement operations, since the operation of both the undulator gap and the c-CCM devices starts only after the devices receive a start command transmitted via the network from the measurement-control PC, it is considered that there is a delay time *t*_1_ between the transmission of the command and the start of operation of the undulator gap and the c-CCM. Since the proposed system could not synchronously acquire real-time undulator-gap values during operation, the value of *t*_1_ was determined through screening to maximize the *I*_0_ photon count. Similarly, fine-tuning of the oscillation period of the c-CCM during actual operation was also performed through screening.

## 3. Results

### 3.1. Evaluation of synchronized scans of undulator and monochromator

**Figure 3:**
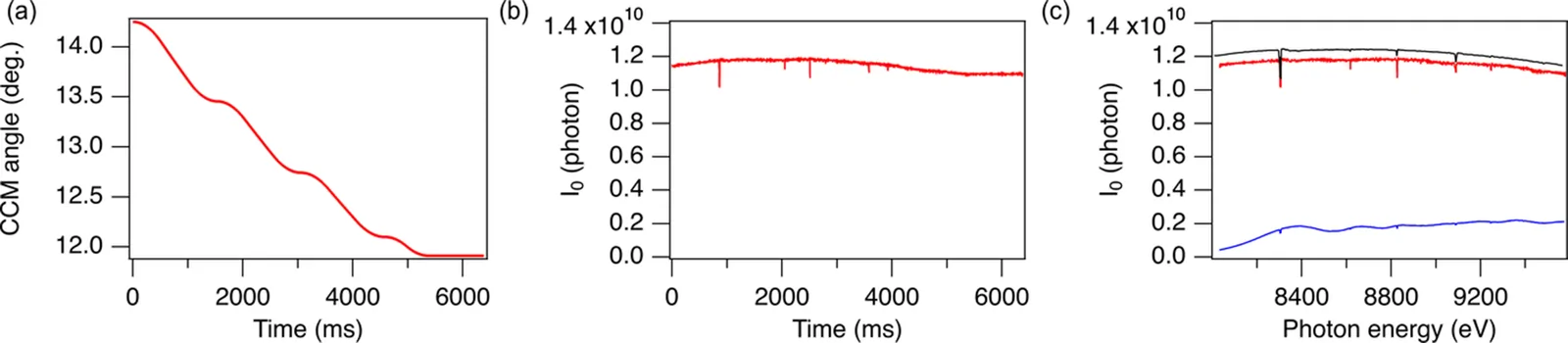
Time profiles of (*a*) the Si(111) c-CCM angle and (*b*) the *I*_0_ photon count at undulator-gap synchronized QXAFS measurement. (*c*) Comparison of *I*_0_ spectra by (black line) step-scan XAFS (1 s exposure per point, photon count scaled to 1/1000), (blue line) tapered and fixed undulator-gap QXAFS (taper: 1.1; measurement time: 6.4 s; sampling rate: 1 kHz), and (red line) undulator-gap synchronized QXAFS (measurement time: 6.4 s; sampling rate: 1 kHz).

In the system constructed at BL36XU, by setting the delay time *t*_1_ between the start of the undulator-gap and c-CCM drive operations to 20 ms [*i.e.* in the control program, the sequence is: (1) undulator-gap drive command, (2) 20 ms wait, (3) c-CCM drive command], we succeeded for the first time in obtaining the changes in c-CCM angle as shown in Fig. 3 ▸(*a*) and the beam intensity (*I*_0_ count) as shown in Fig. 3 ▸(*b*) over a period of 6.4 s. The delay time *t*_1_ = 20 ms is by no means a universal value; rather, it should be considered as a parameter that depends on the specifications of the measurement PC, or the connection status with the other control devices. Still, we obtained good reproducibility of the measurement data for this drive after at least ten repeated measurements. When the beam intensity of the undulator-gap synchronized QXAFS method was normalized by the sampling rate (exposure time) for comparison, it was found to be more than 95% of the beam intensity in the step-scan XAFS method, indicating that the peak energy of the first-order light from the undulator was sufficiently captured by the undulator-gap synchronized monochromator on-the-fly scanning [Fig. 3 ▸(*c*)]. Furthermore, under the same conditions of a 6.4 s spectrum-acquisition time and a 1 kHz sampling rate, it was confirmed that the beam intensity of the undulator-gap synchronized QXAFS method was approximately six times stronger than that of conventional QXAFS measurements using a fixed undulator gap with a taper.

### 3.2. Evaluation of XAFS spectra

#### 3.2.1. Transmission-mode XAFS

**Figure 4:**
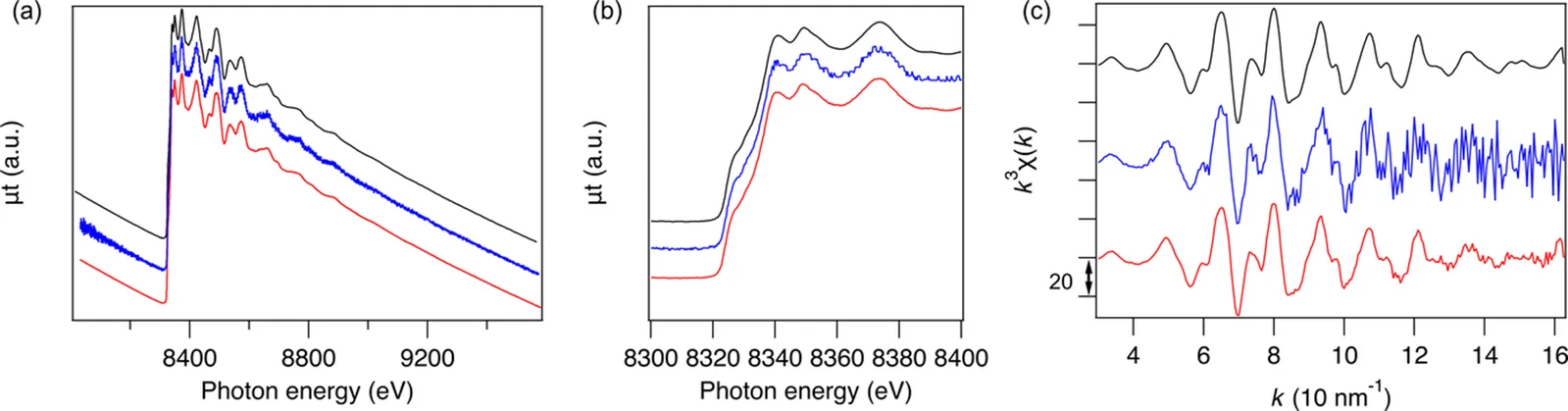
Comparison of Ni *K*-edge XAFS spectra of Ni foil at transmission mode, obtained by (black line) step-scan XAFS (exposure time: 1 s point^−1^), (blue line) tapered and fixed undulator-gap QXAFS (taper 1.1; measurement time: 6.4 s; sampling rate: 1 kHz), and (red line) undulator-gap synchronized QXAFS (measurement time: 6.4 s; sampling rate: 1 kHz). (*a*) Full XAFS spectra, (*b*) XANES spectra and (*c*) *k*^3^-EXAFS oscillation. Data averaging and smoothing over Δ*k* = 0.5 nm^−1^ was applied to raw QXAFS measurement data in (*c*).

Fig. 4 ▸ compares the transmission-mode Ni *K*-edge XAFS spectra of an Ni foil (thickness: 5 µm) obtained using three measurement methods: step-scan XAFS, QXAFS with a tapered and fixed undulator gap, and undulator-gap synchronized QXAFS. With increased beam intensity, the XANES spectral data obtained using the undulator-gap synchronized QXAFS method showed a clear improvement in signal-to-noise ratio (SNR) compared with QXAFS data measured with a tapered fixed undulator gap. Furthermore, the quality of the data in the XANES region was nearly indistinguishable from that of step-scan XAFS with an exposure time of 1 s point^−1^. Since QXAFS measurements are performed at a high sampling rate of 1 kHz, averaging and smoothing multiple measurement points for the EXAFS region over a range of approximately Δ*k* = 0.5 nm^−1^ further improved the SNR of the EXAFS oscillations, and it was found that the EXAFS oscillation data obtained from undulator-gap synchronized QXAFS are of a quality comparable to those from step-scan EXAFS.

#### 3.2.2. Partial fluorescence yield (PFY) mode XAFS

**Figure 5:**
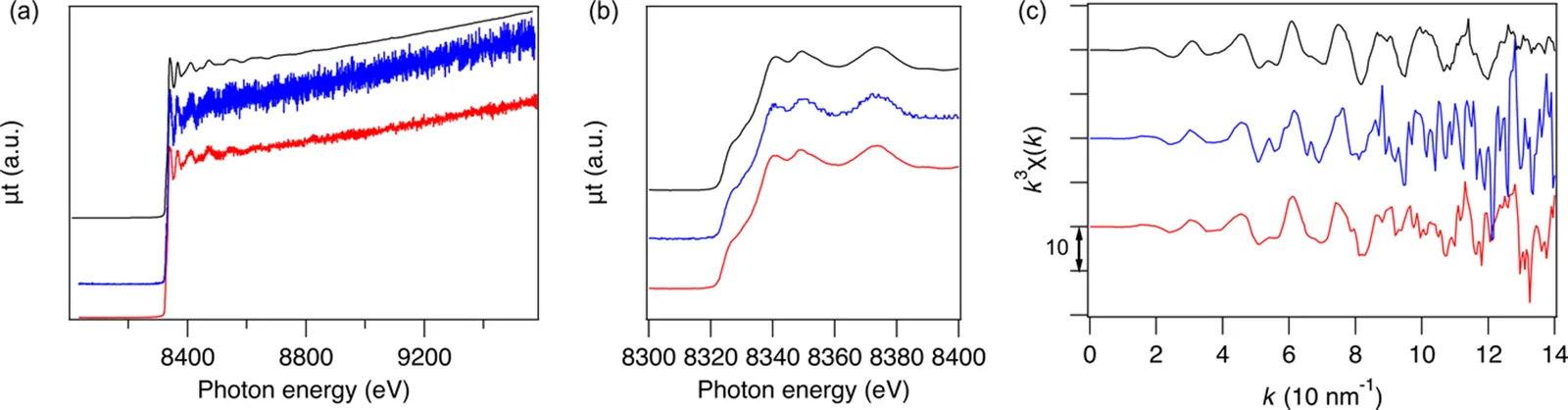
Comparison of Ni *K*-edge XAFS spectra of Pt_3_Ni/C electrocatalysts in the MEA of the PEFC at PFY mode, obtained by (black line) step-scan XAFS (exposure time: 1 s point^−1^), (blue line) tapered and fixed undulator-gap QXAFS (taper: 1.1; measurement time: 6.4 s; sampling rate: 700 Hz), and (red line) undulator-gap synchronized QXAFS (measurement time: 6.4 s; sampling rate: 700 Hz). (*a*) Full XAFS spectra, (*b*) XANES spectra and (*c*) *k*^3^-EXAFS oscillation. Data averaging and smoothing over Δ*k* = 0.5 nm^−1^ was applied to raw QXAFS measurement data in (*c*).

Fig. 5 ▸ shows a comparison of the PFY-mode *K*-edge XAFS spectra of Pt_3_Ni/C electrocatalysts in the MEA of the PEFC, obtained using three different measurement methods: step-scan XAFS, QXAFS with a tapered and fixed undulator gap, and undulator-gap synchronized QXAFS. The MEA (prepared by Eiwa Co., Ltd., Japan) contains a Nafion NR-212 membrane (Sigma–Aldrich; thickness: 50 µm), a Pt_3_Ni/C cathode electrocatalyst [TECNiE52, Tanaka Kikinzoku Kogyo (TKK) Co., Ltd., Japan; Pt loading: 0.33 mg cm^−2^; Ni loading: 0.037 mg cm^−2^] and a Pt/C anode electrocatalyst (TEC10E50E, TKK; Pt loading: 0.33 mg cm^−2^). The edge jump in the transmission-mode Ni *K*-edge XAFS spectra of the MEA sample is estimated to be Δμ*t* = 0.01. The quality of the XANES spectral data obtained using the undulator-gap synchronized QXAFS method was comparable to that obtained with step-scan XAFS at an exposure time of 1 s point^−1^. Furthermore, the SNR was substantially improved compared with QXAFS measurements obtained with tapered or fixed undulator gaps. In the EXAFS region, when averaging and smoothing were applied over a range of approximately Δ*k* = 0.5 nm^−1^, oscillation data from the undulator-gap synchronized QXAFS exhibited higher SNR than the QXAFS measurement under a tapered undulator gap, with the same spectral time resolution. This indicates that the EXAFS oscillation data can be used for local coordination structure analysis up to higher *k* ranges.

## 4. Discussion

**Figure 6:**
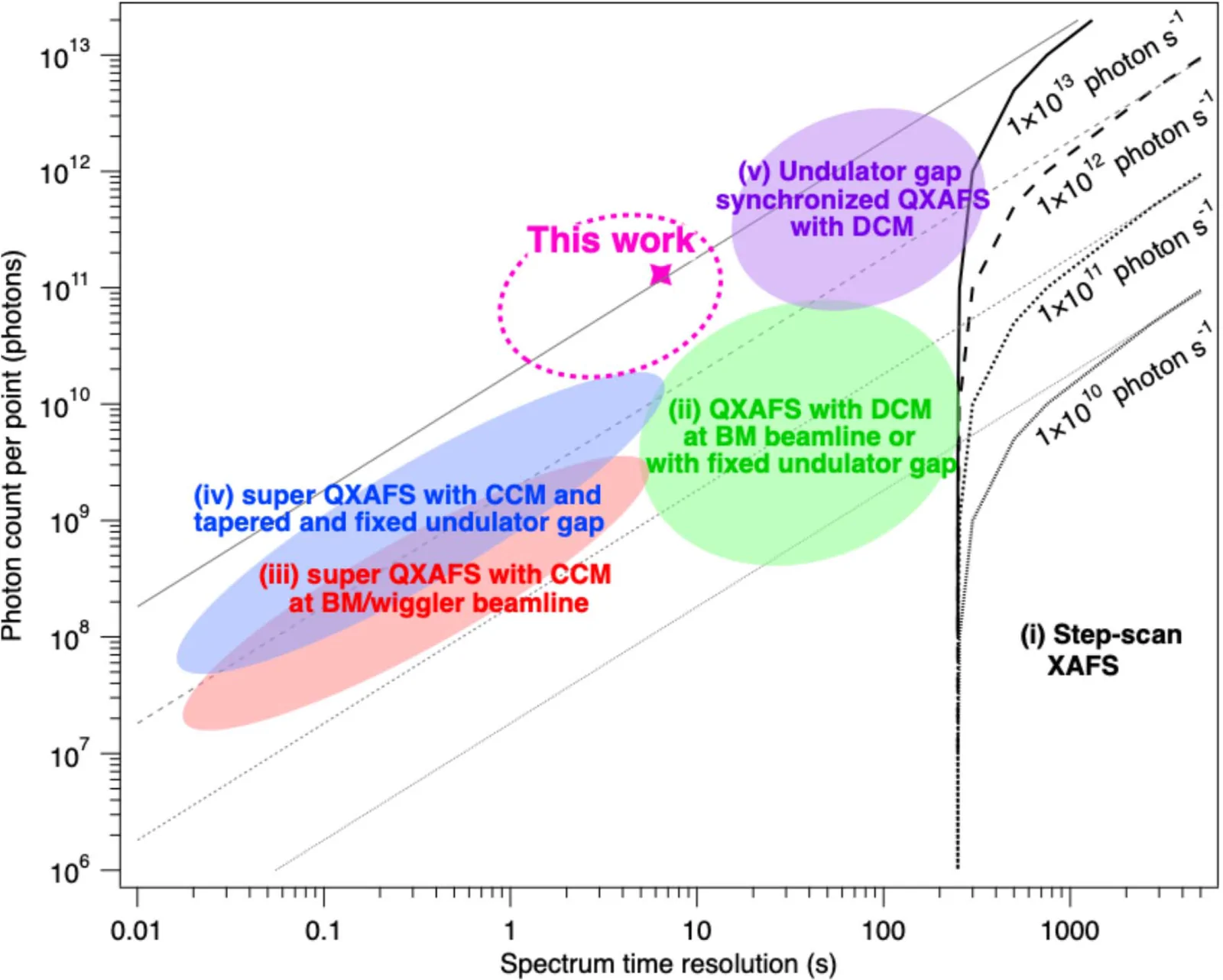
Relationship between the measurement time required to acquire an XAFS spectrum in extension of the EXAFS region (spectrum time resolution) and the photon count per point, for different beamlines and measurement types. The photon-energy range is 8.03–9.57 keV (Ni *K* edge), with 530 points, and the photon count per point is normalized to match the number of points by step-scan XAFS. The region (i) in the figure assumes that there is an overhead of 0.5 s per spectral-measurement point during the monochromator and undulator-gap scanning when the incident X-ray flux is in the range of 1 × 10^10^–1 × 10^13^  photons s^−1^. The regions (ii)–(v) in the figure are classified based on the following references: (ii) QXAFS with DCM at bending magnet (BM) beamlines, or with fixed undulator gap (Nomura *et al.*, 2007 ▸; Dent *et al.*, 2009 ▸; Uruga *et al.*, 2009 ▸; Liu *et al.*, 2012 ▸; Okajima *et al.*, 2013 ▸; Mathon *et al.*, 2015 ▸; Tabuchi *et al.*, 2016 ▸; Chu *et al.*, 2017 ▸); (iii) super QXAFS with CCM at BM/wiggler beamlines (Stötzel *et al.*, 2008 ▸; Khalid *et al.*, 2011 ▸; Müller *et al.*, 2016 ▸); (iv) super QXAFS with CCM, and tapered and fixed undulator gap (Nonaka *et al.*, 2012 ▸; Ishiguro *et al.*, 2014 ▸, 2016 ▸; Ozawa *et al.*, 2018 ▸; Caliebe *et al.*, 2019 ▸; Bornmann *et al.*, 2019 ▸; Uruga *et al.*, 2019 ▸); and (v) undulator-gap synchronized QXAFS with DCM (Krempaský *et al.*, 2010 ▸; Izquierdo *et al.*, 2012 ▸; Chernikov *et al.*, 2016 ▸; Hidas *et al.*, 2022 ▸). The present work corresponds to the pink star, achieving both the shortest acquisition time and the highest photon flux among reported undulator-gap synchronized QXAFS systems.

Fig. 6 ▸ shows the relationship between the acquisition time for XAFS spectra (including the EXAFS region) and the number of photons incident per point in the spectrum, as a function of beamline and measurement method. Using the step-scan XAFS method, even on beamlines with the strongest X-ray intensity, an overhead of ∼0.5 s was expected for the operation of the monochromator and the gap (undulator beamline) at a single energy point. Therefore, regardless of how short the exposure time per point is made, the total acquisition time remains at least ∼250 s [Fig. 6 ▸(i)]. QXAFS enables EXAFS measurements in tens of seconds to sub-minutes through on-the-fly operation of the monochromator [Fig. 6 ▸(ii)]. The introduction of super QXAFS using a CCM enables EXAFS measurements faster than the previous maximum spectral-measurement time of tens of milliseconds [Figs. 6 ▸(iii) and 6 ▸(iv)]. However, in undulator beamlines, to accommodate a wide range of measurement photon energies extending to 1 keV, a taper was introduced into the undulator, sacrificing maximum photon flux as a trade-off [Fig. 6 ▸(iv)]. Conversely, undulator-gap synchronized QXAFS has enabled EXAFS measurements with higher photon fluxes over timescales ranging from tens of seconds to sub-minutes [Fig. 6 ▸(v)]. In this study, by utilizing the high speed and driving flexibility of the CCM to achieve undulator-gap synchronized QXAFS, we achieved a measurement time of 6.4 s and a photon flux of the order of 1.2 × 10^13^ photons s^−1^ (1.4 × 10^11^ photons per energy point, if averaged to 530 energy points) (pink star in Fig. 6 ▸). This indicates that measurements can be performed several times faster than with conventional feedback-type undulator-gap synchronized QXAFS, and that the flux is six times stronger than that of tapered- and fixed-gap QXAFS with the same measurement time.

Signal-to-baseline ratio (SBR) for both transmission- and fluorescent-mode XAFS is proportional to 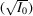. This means that the SBR is improved by approximately a factor of 2.5 in the undulator-gap synchronized QXAFS system, compared with tapered and fixed undulator-gap QXAFS. On the other hand, because XAFS spectra contain background components, the SNR is usually lower than the SBR (SNR < SBR). However, when background components are removed, as in PFY mode, the SNR value approaches the SBR (SNR ≃ SBR), reflecting the greater improvement in the *I*_0_ flux compared with transmission mode. EXAFS oscillation χ(*k*) is given with the following equation by plane-wave scattering theory,
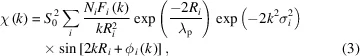
where 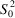 is the intrinsic loss factor, λ_p_ is the mean free path of photoelectrons, and *N*_*i*_, *R*_*i*_, 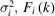 and ϕ_*i*_(*k*) are the coordination number, interatomic distance, Debye–Waller factor, back-scattering amplitude and phase shift of the *i*th shell, respectively. Thus, the SNR of oscillation amplitude is governed by the term 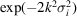. Here, we assume that the Debye–Waller factor value is 5 × 10^−5^ nm^2^, which is the typical value for metal–metal bonds, and set the upper limit of the analyzable *k* ranges in the EXAFS oscillations obtained from the tapered and fixed undulator-gap QXAFS measurement to ∼100 nm^−1^, as shown in Fig. 5 ▸(*c*). When the *I*_0_ flux and SNR of the QXAFS measurement are improved by a factor of 2.5, the upper limit of the analyzable *k* ranges in the EXAFS oscillations will be extended from *k* = 100 nm^−1^ to *k* = 138 nm^−1^. This estimation is in close agreement with the upper limit of the analyzable *k* range for the EXAFS oscillations obtained from the undulator-gap synchronized QXAFS measurements shown in Fig. 5 ▸(*c*).

Currently, we have only demonstrated undulator-gap synchronized QXAFS in the forward scan (from lower photon energy to higher) for single times. To achieve two-way (forward and reverse) and repetitive undulator-gap synchronous energy scans, it is necessary to send a new command to the undulator to start scanning in the reverse direction soon after the forward/reverse scan is complete, which requires an interval of ∼0.1–0.2 s. However, the motion of the c-CCM can be reproduced by its vibrational behavior, including this interval time, and there are almost no technical difficulties in extending this to time-resolved measurements. Another technical challenge is that the temporal resolution is limited by the drive frequency of the undulator gap. Under the standard undulator-gap drive scheme at the current SPring-8, the system has operated with step-like behavior, involving repeated trapezoidal drives in 0.5 mm increments. However, since there are no particular constraints on light-source optics control for moving in a single trapezoidal drive, further slight improvements in spectral-measurement time are expected through optimization of the gap drive. Furthermore, since a new IVU, expected to have improved undulator-gap drive speed, is scheduled to be implemented as part of the ongoing upgrades to SPring-8-II, the spectral time resolution of QXAFS is expected to improve further with this technique. We should also care about the effect of the spatial distribution by c-CCM scanning. In this study, we focused on samples — such as pellets or solutions — that can be considered macroscopically homogeneous. However, since the CCM is not a monochromator that guarantees fixed emission position, we must take this energy dependence of the incident X-ray optical axis height into account in future studies involving inhomogeneous sample systems and spectro-microscopic measurements. For example, the channel distance (*D*) of the c-CCM at SPring-8 BL36XU is about 3.1 mm. The vertical displacement *H* of the optical axis between the incident and emitted X-rays when the c-CCM is at an angle θ is given by
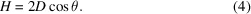
Therefore, for X-rays emitted from the Si(111) c-CCM at the Ni *K*-edge EXAFS range (8.03–9.57 keV), a vertical optical axis shift of approximately Δ*H* = 57 µm is expected. In that case, we should consider approaches to compensate for the optical axis variation, such as an additional feedforward control system to have the sample stage track the monochromator motion.

Future QXAFS measurements using this technique are expected to cover the area indicated by the pink dotted line in Fig. 6 ▸. As shown in Figs. 4 ▸ and 5 ▸, the spectral quality has sufficiently improved even in transmission-mode measurements. Although this method dramatically increases the number of cycles in the undulator gap (with an expected annual total of over 150 000 cycles), and while we must consider the impact on its durability, it enables *operando* time-resolved measurements of the order of seconds for various dilute sample reaction systems. For example, it can be used to monitor the reactions/dissociations of minor metal species from electrodes to electrolyte solutions, or the kinetics of electrocatalysts in practical PEFCs for automotive applications, with MEAs containing a metal loading of 0.1 mg cm^−2^ or less. Previously, such measurements took minutes using fluorescence XAFS. This method enables the determination of the local coordination structure of trace elements in such samples within a matter of seconds, making it possible to capture structural changes during electrochemical reactions in real time. Furthermore, the proposed technique is not limited to simple XAFS measurements; it can be relatively extended to emission spectroscopy (HERFD-XANES and RIXS) and spectro-microscopy [XAFS-CT (Matsui *et al.*, 2022 ▸), TXM-XAFS, spectro-ptychography (Hirose *et al.*, 2020 ▸), *etc*.]. Consequently, it is expected to serve as a foundational technology that significantly contributes to the *operando* and high-throughput capabilities of advanced X-ray measurement techniques.

## Data Availability

The data supporting the results reported in the article are available upon request.

## Appendix A Derivation of the relationship between the undulator gap and the fundamental wavelength (energy)

The strength of the magnetic field between the magnets in an undulator (*B*) is given by the following relationship with the gap distance (Δ*g*_u_),

where λ_u_ denotes the period length of the magnets and α is a constant. The *K* value of the undulator is given by the following equation,

where *e* denotes the elementary charge, *m*_e_ is the mass of an electron and *c*_0_ is the speed of light in a vacuum. The peak wavelength of the X-ray spectrum emitted from the undulator or the first-order wavelength, λ_peak_, along the undulator axis (θ = 0), is given by the following equation,

where γ denotes the Lorentz factor. Using equations (5)–(7), the relationship between the undulator gap (Δ*g*_u_) and the peak energy (*E*_peak_) is expressed as follows,

where constants *a*, *b* and *c* are given as

and
